# A Machine Learning Approach for High-Dimensional Time-to-Event Prediction With Application to Immunogenicity of Biotherapies in the ABIRISK Cohort

**DOI:** 10.3389/fimmu.2020.00608

**Published:** 2020-04-07

**Authors:** Julianne Duhazé, Signe Hässler, Delphine Bachelet, Aude Gleizes, Salima Hacein-Bey-Abina, Matthieu Allez, Florian Deisenhammer, Anna Fogdell-Hahn, Xavier Mariette, Marc Pallardy, Philippe Broët

**Affiliations:** ^1^Research Center, Ste-Justine Hospital, Montreal, QC, Canada; ^2^UMR 1018, INSERM, CESP, Paris-Saclay University Faculty of Medicine, Paul-Brousse Hospital, Villejuif, France; ^3^UMR 959, INSERM, Immunology-Immunopathology-Immunotherapy (i3), Sorbonne University, Paris, France; ^4^Biotherapy (CIC-BTi), Pitié-Salpêtrière Hospital AP-HP, Paris, France; ^5^CIC-EC 1425, INSERM, Department of Biostatistical Epidemiology and Clinical Research, Bichat Hospital, Assistance Publique-Hôpitaux de Paris AP-HP Nord, Paris, France; ^6^Clinical Immunology Laboratory, Le Kremlin-Bicêtre Hospital AP-HP, Paris-Saclay University, Le Kremlin-Bicêtre, France; ^7^UMR 996, INSERM, Faculty of Pharmacy, Paris-Saclay University, Châtenay-Malabry, France; ^8^UTCBS, CNRS UMR 8258, INSERM U1022, Faculty of Pharmacy, Paris-Descartes-Sorbonne-Cité University, Paris, France; ^9^Department of Gastroenterology, Saint-Louis Hospital, AP-HP, Paris-Diderot University, Paris, France; ^10^Innsbruck Medical, Innsbruck, Austria; ^11^Department of Clinical Neuroscience, Karolinska Institutet, Stockholm, Sweden; ^12^UMR 1184, INSERM, Centre for Immunology of Viral Infections and Autoimmune Diseases, Paris-Saclay University, AP-HP Université Paris-Saclay, Paris, France

**Keywords:** immunogenicity, biotherapy, machine learning, survival random forest, prediction

## Abstract

Predicting immunogenicity for biotherapies using patient and drug-related factors represents nowadays a challenging issue. With the growing ability to collect massive amount of data, machine learning algorithms can provide efficient predictive tools. From the bio-clinical data collected in the multi-cohort of autoimmune diseases treated with biotherapies from the ABIRISK consortium, we evaluated the predictive power of a custom-built random survival forest for predicting the occurrence of anti-drug antibodies. This procedure takes into account the existence of a population composed of immune-reactive and immune-tolerant subjects as well as the existence of a tiny expected proportion of relevant predictive variables. The practical application to the ABIRISK cohort shows that this approach provides a good predictive accuracy that outperforms the classical survival random forest procedure. Moreover, the individual predicted probabilities allow to separate high and low risk group of patients. To our best knowledge, this is the first study to evaluate the use of machine learning procedures to predict biotherapy immunogenicity based on bioclinical information. It seems that such approach may have potential to provide useful information for the clinical practice of stratifying patients before receiving a biotherapy.

## Introduction

In recent years, the introduction of biopharmaceuticals products (BPs) has opened a new area in the treatment of various cancer and auto-immune diseases. However, for some patients, the therapeutics induce an activation of the immune system, leading to the formation of anti-drug antibodies (ADA). These ADA may lead to partial or complete loss of efficacy of the drug (1). The mechanisms suspected for being involved in the immunogenicity of biotherapies process are patient-related (genetic background, immunological status, prior exposure, prior disease, co-administered drugs) or treatment-related (drug characteristics and formulations, route, dose, frequency of administration) but their relative contributions to the development of ADAs is not fully understood and remains to be deciphered for being used for predictive purpose (1, 2).

In this context, the IMI-funded ABIRISK consortium (3) had set up a real-world observational multicohort of patients suffering from various auto-immune diseases such as multiple sclerosis, rheumatoid arthritis, Crohn's disease, and ulcerative colitis. Participants recruited in the study were naive for the biotherapies they received during the study and were monitored during 12 months. ADA concentration was first measured at baseline and then at defined timepoints. The investigated BPs were TNF inhibitors, IFNβ, anti-CD20, and anti-IL6R. For each subject, the time-to-occurrence of ADA provided a way to evaluate both the propensity to produce or not ADA (termed in the following as being an “immune-reactive” or “immune-tolerant” subject) and the dynamic of the ADA production (early/late) among the immune-reactive subjects. The main objective was to provide an estimate of the probabilistic susceptibility of an individual to produce ADA based on the drug received and the subject's clinical and genetic information.

With the production of high-dimensional datasets (so-called big data) there is nowadays a growing interest in using machine learning (ML) approaches for clinical prediction (4). Indeed, ML is particularly appealing for situations where complex non-linear relationships are expected to play a key role into the disease process such as in biotherapy immunogenicity. Random Forests (RF) as introduced in the seminal paper of Breiman (5) is one of the most effective ML approaches for prediction. Thus, RF and its variants are more and more frequently considered for delivering big-data-driven clinical prediction algorithms. Broadly speaking, the RF builds a series of decision trees from which a final prediction is obtained by combining the predictions from each individual tree. These latter tree-based learners are non-parametric approaches that partition recursively the space of predictor variables into disjoint sub-regions (so-called terminal nodes or leaves) that are homogeneous according to the outcome of interest. These partitions are obtained from a splitting criterion that either minimizes the within-node heterogeneity or maximizes the between-node heterogeneity (6). The well-known instability of each individual tree-based structure has been the main motivation to the development of RF, the main idea being that the combination of several survival tree predictors has better predicting power than each individual tree. In the original RF procedure proposed by Breiman (5) each tree is built using a random set of individuals with replacement (bootstrapping) and each split of the tree is evaluated on a random small subset of predictor variables. The main goal of this process is to increase the diversity of the tree-based learners that are aggregated at the end. Among the key features of the random forests, the random choice of both the individuals and the features together with the splitting criterion play critical roles. Since the first introduction of RF, tree-based learners have been extended to censored data (termed survival trees) (7) and integrated in RF framework [termed as random survival forests (RSF)] (8, 9).

For this immunogenicity prediction study, we have been confronted to some specific issues that prompted us to consider a modified RSF approach. We first had to cope with a situation where we had collected a huge set of candidate predictors (clinical and genetic markers) but only a small number were expected to be relevant for prediction. Secondly, we studied a mixed population of subjects with both susceptibles (immune-reactive) and non-susceptibles (immune-tolerant) subjects for the outcome of interest (ADA occurrence). The first issue is linked to the RF procedure where at each split of a tree the recursive partitioning process is only applied to a random subset of all the predictors. Thus, when the number of relevant predictors is overwhelmingly small as compared to the size of the non-relevant ones (noise), the randomness in the variables selection leads to most of the subspaces having weak predictive accuracy and thus affects the final prediction of the RF. The second issue relates to the fact that our studied population is a mixture of immune-tolerant and immune-reactive subjects. The immune-tolerant subjects are those whose immune system is in a state of unresponsiveness to the exposure of the drug and thus will not produce ADA whereas the immune-reactive subjects are those who are able to produce detectable levels of antibodies. In such mixed population (10), the logrank statistic which is the commonly used splitting criterion for RSF does not take into account the dynamic of the ADA production which may decrease its discriminative performance.

In order to cope with these issues, we have considered a strategy which relies upon a particular splitting criterion and uses a modified RSF strategy with a random subspace sampling step. The splitting criterion is related to a previous work on heterogeneous population with non-susceptible patients (11). The random subspace sampling strategy follows the proposal of Panov and Dzeroski (12), that combines bagging (random subsamples with replacement) and random subsampling (random subspaces).

In this paper, we first present our modified RSF procedure and then apply this latter for predicting the occurrence of anti-drug antibodies from the ABIRISK cohort.

## Materials and Methods

###  Material

These data come from a real-world observational prospective multicenter cohort of patients suffering from various auto-immune diseases. Patients who had been prescribed a biotherapy by a physician were followed for 12 months. The choice of the treatment was left to the physician.

Clinical data were recorded into an electronic Case Report Form. DNA samples and serum samples were collected for genetic analyses and ADA testing, respectively. Serum samples for ADA testing were collected at baseline before start of BP therapy and subsequently at each study visit after start of therapy. Anti-drug antibodies were detected by specific validated assays for each BP and analyzed in central ABIRISK laboratories.

Patients were followed for 12 months from the start of the therapy. The time-to-event (ADA positivity) was defined as the period of time from the date of first treatment to the time of first ADA positivity. Patients without ADA occurrence were censored at the date of their last follow-up (drop-out, drug switch), or administrative censoring (12 months). Patients with their consent for genetic testing, available high-quality blood DNA samples were selected for genotyping. Among the 609 recruited patients, 560 were eligible and 501 DNA samples were available.

Genotyping was performed with Infinium OmniExpress-24 v1.2 BeadChip. This array interrogated over 700,000 genetic markers (single nucleotide polymorphisms, SNPs) located throughout the genome. We imputed the missing SNPs using the Michigan Imputation Server with a European population panel as reference (13). After quality control procedures, we had 457 individuals and 495,792 SNPs. For the predictive analysis, in order to avoid highly imbalanced groups, we focused on common SNPs with intermediate minor allele frequencies (MAF > 25%). Thus, we retained 287,611 SNPs. Among these 457 patients, 114 were treated with Adalimumab, 76 with Infliximab, 64 with Etanercept, 27 with Rituximab, 35 with Tocilizumab, 64 with IFNβ-1a sub-cutaneous, 40 with IFNβ-1b sub-cutaneous, 37 with IFNβ-1a intra-muscular. The clinical variables considered for the predictive analysis were biotherapy, disease, age, sex, tobacco smoking, body mass index, previous or concomitant medications. For the missing clinical variables, we considered a basic imputation strategy where we replaced the missing values by the mean of the non-missing values (continuous variable) or the most common class (binary variable).

For the predictive analysis, the clinical variables were included without recoding for binary variables (sex, tobacco smoking, previous or concomitant medications) and continuous variables (age, body mass index). For unordered categorical variable (disease), we considered the four partitions as candidate variables. For the treatment variable and based upon previous analyses, we considered three immunogenicity groups. The low immunogenicity group (Etanercept, IFNβ-1a i.m.), the intermediate immunogenicity group (Tocilizumab, Infliximab and IFNβ-1a s.c.) and the high immunogenicity group (Rituximab, Adalimumab, IFNβ-1b s.c.). In practice, we included the treatment as an ordered variable (low/intermediate/high immunogenicity level) and also as dummy variables (one drug vs. the others). Genotyping data were considered as ordered variables (based on the number of alternative variants) such as the partition explored recessive and dominant genetic effects for the alternative variant.

###  Methods

#### Survival Model

##### Notations

Let denote *T* the time-to-ADA detection and *C* the censoring time. For each subject *i* (*i* = 1, …*n*), *X*_*i*_ = *min*(*T*_*i*_, *C*_*i*_) is the observed time of follow-up and δ_*i*_ = 1_(_*X*__*i*_ = *T*_*i*_)_ the indicator of ADA detection (positivity). We also denote *Y*_*i*_(*t*) = 1_(*t* ≤ _*X*__*i*_)_ the indicator of being at risk for the event at time *t*. Let *G*1_*i*_ = (*G*1_*i*1_, ⋯ , *G*1_*iq*_) be the *q*-dimensional vector encoding treatment and clinical variables. All these variables were binary or categorized variables. Let *G*2_*i*_ = (*G*2_*i*1_, ⋯ , *G*2_*ip*_) be a *p*-dimensional vector of genotypes for a patient *i*. Here, the genotype information relying upon *p* biallelic genetic markers (SNPs). The genotype of subject *i* is coded as an ordinal 0;1;2 variable where the values represent the number of alternative variants of the subject. Finally, let *G*_*i*_ = (*G*1_*i*_, *G*2_*i*_). This *m*-dimensional (*m* = *p* + *q*) vector gathers information from the treatment, the clinic and the genotype of each subject.

When building each individual tree, at each node *h*, for each of the *m* variables of the *G*_*i*_ vector, the process searches for the best binary split.

##### Mixture Model

In this work, we take into account that the population under study is a mixture of immune-reactive and immune-tolerant patients. Here, the immune-reactive group is composed by those who are susceptible to produce detectable levels of antibodies within the 1-year window of monitoring. The immune-tolerant group is composed by those who are immune-tolerant to the BPs that is to say that they will not produce detectable levels of antibodies. As both immune-reactive and immune-tolerant subjects cannot be distinguished in the censored subset, we had to consider long-term survival models that explicitly consider the existence of a proportion of immune-tolerant subjects.

For modeling survival data with a proportion of non-susceptible individuals, there are broadly two mains frameworks. The first one relies on two-component mixture models whereas the second one relies on defining the cumulative hazard as a bounded increasing positive function (10, 14). In this paper, we consider the latter framework since it has some interesting mechanistic interpretation of the biological mechanism of the occurrence of the event of interest. More precisely, we propose to model the distribution of the time-to-ADA detection through a simplified mechanistic model whereby each individual may or may not be able to produce ADA in response to the introduction of the biotherapy. This model is related to a previous work on long-term survival model with application to clinical oncology (11).

Here, we consider that ADA are produced by the activation of unobservable BP-specific (T-dependent) B-cell clones that emerge and become immunocompetent ADA-producing clones. Positivity occurs as soon as any one of the B-cell clones is able to produce levels of ADA of sufficient affinity and titre for being detected by the assay. Thus, the observed time-to-detection is the first time-to-detection associated with a competent B-cell clone. If no competent B-cell clone is produced by an individual, then the patient is considered as immune-tolerant and his/her time-to-detection is considered, theoretically, as the infinity.

Since the B-cell clones are not directly observed for each individual, we cannot obviously specify the individual survival distribution. However, if we assume a particular distribution for the number of unobserved B-cell clones, we can specify the marginal or population (averaged over the population under study) survival function. Assuming a Poisson distribution for the number of B-cell clones, we can obtain the population survival distribution with bounded cumulative model that is used in this article and presented just below (11, 15).

At each node, for each binary split candidate variable *W*_*k*_ = 0, 1 (*k* = 1, …, *K*), we consider the following population survival distribution:

(1) $$\begin{array}{l} S\left(t|W_{k}=w\right)=\exp\{-\Lambda \left(t|w)\right\}=\exp\{-\theta \left(w\right)\left[1-H\left(t|w\right)]\right\} \end{array}$$

Here, *H*(*t*|*w*) is an unspecified continuous positive function increasing from zero to infinity that is similar to a cumulative hazard risk function and θ(*w*) is a positive quantity. Thus, *S*(*t*|*w*) shows a tail defect (related to the fraction of immune-tolerant subjects) with *S*(*t* = ∞|*w*) = exp[−θ(*w*)] > 0. The cumulative hazard function Λ(*t*|*w*) = −*log*(*S*(*t*|*w*)) is bounded by θ(*w*).

In the following we will consider a classical multiplicative structure such as: $\theta \left(w\right)=\theta_{0}e^{\alpha w}$ and $H\left(t|w\right)=H_{0}\left(t\right)e^{\beta w}$ where θ_0_ and *H*_0_(*t*) are the baseline tail defect and pseudo-cumulative hazard function, respectively, and α, β are unknown parameters. Here, α quantifies the difference of the immune-tolerant fractions between the two groups and β quantifies the difference between the two groups in the dynamic of the production of ADA among the immune-reactive subjects. If α = 0 there is no difference between the immune-tolerant fractions of the two groups. If β = 0 the dynamic of ADA production among immune-susceptible patients are identical for the two groups.

At any split, the hazard ratio between the two groups is such as:

(2) $$\begin{array}{l} \frac{\lambda \left(t,W_{k}=1\right)}{\lambda \left(t,W_{k}=0\right)}=e^{\alpha }e^{\beta }e^{-H_{0}\left(t\right)\left(e^{\beta }-1\right)} \end{array}$$

with λ(*t, W*_*k*_ = 0) and λ(*t, W*_*k*_ = 1), the instantaneous rates of ADA detection for group 0 and 1. As seen above, due to the existence of an immune-tolerant faction, the hazard ratio is not constant over time (non-proportional relationship).

#### Splitting Criterion

In the spirit of classical partial-likelihood-based splitting criteria (e.g., the logrank statistic), we propose to use a criterion which is based upon the score vector derived under the survival model presented above and computed under the null hypothesis of no difference between the two groups for both the immune-tolerant fraction and the dynamic of ADA production among immune-reactive patients. This criterion aims to identify the splitting candidate variable which maximizes the between-node heterogeneity. Maximizing the between-node heterogeneity leads to minimize the variability inside each node. In other words, it leads to more homogeneous groups with respect to the outcome of interest. This criterion is identical to the global test proposed by Broët et al. (11)

#### Modified Random Survival Forests Procedure

In this section, we first recall the basic principles for building a survival tree and then we present the proposed procedure.

##### Building a survival tree

In practice, a survival tree is grown by first splitting the whole dataset (so-called the root node) into two so-called child nodes that maximize between-node heterogeneity. The same procedure is then repeated for each child node until each node reaches a predetermined stopping rule (e.g., minimum node size) or be homogeneous. A node that cannot be split any further is called a terminal node (or leave). Each terminal node is a distinct partition of the sample which is characterized by a unique combination of the predictors.

**Figure 1:**
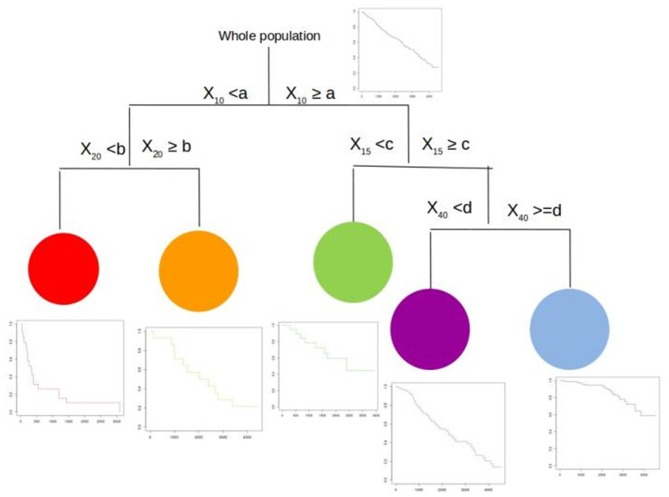
A survival tree.

In Figure 1, we present an example of a survival tree. Here, we start with the whole population (top). Then, the procedure searches for the best splitting variable (which maximizes our splitting rule). Here, the process selects the variable *X*_10_ with threshold cutpoint *a* which splits the population in two sets of individuals, those with *X*_10_ < *a* (left) and those with *X*_10_ ≥ *a* (right). For each of these two groups, the algorithm now searches for the next best splitting variables. On the left, the procedure selects the variable *X*_20_ with threshold *b*. On the right, the procedure selects the variable *X*_15_ with threshold *c*. For each of these four groups, the process continues to split in a binary fashion or stops if it has reached a predetermined stopping rule. As seen on the figure, on the left side, it stops. On the right side, it splits the far-right group in two new groups based upon the variable *X*_40_ with threshold *d*. Now, the algorithm stops as it reached a predetermined stopping rule.

We end up with a population with five more homogeneous groups (with their respective survival curves). These five groups are defined by a combination of the four splitting variables with their corresponding cutpoints. For each group, the Nelson–Aalen or Kaplan–Meier estimates of the survival function is calculated. It is worth noting that the use of the Nelson–Aalen or Fleming–Harrington estimator (16) (using the cumulative hazard) would be more suited for leaves with a small number of subjects rather than the Kaplan–Meier (product-limit) estimator. Here, the group in red has the worst prognosis whereas the group in blue has the best prognosis. The others have intermediate prognosis.

##### Building a modified Random Survival Forest with random subspace sampling

Our dataset (noted L) consisting of *n* independent individuals with observed outcomes and predictor variables such as $L=\left\{\left(X_{i},\delta_{i},G_{i}\right),\;i=1,\ldots ,n\right\}$. The proposed procedure is based on the following algorithm.

We first draw with replacement *n* individuals of our dataset L and thus create a bootstrap sample (denoted $L_{b}$ with *b* = 1, …, *B*) from the original dataset. This means that in average 63% of the original sample is included in the bootstrap sample and 37% are left out. At the same time, we randomly select a subspace sample of the predictors (denoted $P_{b}$) of dimensionally p^*^ (with p^*^ < *m*).

Based on this subspace bootstrap sample $L_{b}^{P_{b}}$, we build a survival tree and repeat the process *B* times as described just below.

• Build a survival tree from $L_{b}^{P_{b}}$.
* For each split candidate variable *W*_*k*_, we compute the corresponding splitting criterion *S*(*W*_*k*_) presented above. We do the same procedure for all the split candidate variables.
* Then, we find the best split S^*^ which is the one having the maximum value over all the candidates. Then, a new node is built and the observations are splitted accordingly.
* We iterate the process until each node reaches a pre-defined minimum node size or be homogeneous.
* We construct the final tree denoted $T_{b}^{P_{b}}\left(W^{b}\right)$ where $W_{l}^{\left(b\right)}$ (*l* = 1, …, *L*(*b*)) is a vector of indicator variables representing the *L*(*b*) leaves of the tree such that $W_{il}^{\left(b\right)}=1$ if the *i*^*th*^ observation belongs to the *l*^*th*^ terminal node of $T_{b}^{P_{b}}$, and 0, otherwise.
• Repeat the process *B* times.

At the end of the process, we have a series of *B* survival trees known as a random survival forest.

**Figure 2:**
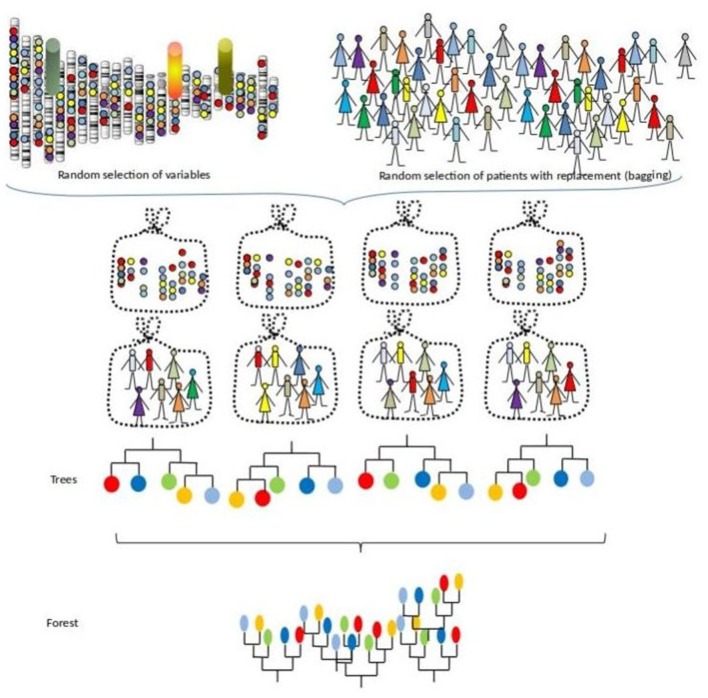
A survival forest.

This procedure can also be seen as a bootstrapped RSF with random subsampling of the feature space. Figure 2 illustrates this process with iterations of building survival trees from bagged samples and random subspace sample of the predictors.

##### Evaluation of the Predictive Accuracy

Here, each survival tree is built from a bootstrap sample of the data. The individuals who are in this sample are called “in-bag” individuals and those who are not are called “out-of-bag” individuals. Thus, we can compute the survival predictions of each “out-of-bag” individual. In practice, the split variables of the patient are dropped down the tree until it reaches a terminal node. Then, the patient's survival prediction is the estimated survival of this terminal node. It is a valid prediction which is not optimistically biased since the survival is predicted using a tree that do not use this individual. The final prediction are given by averages overall the estimates. Then, we can compare survival predictions to what was really observed.

**Figure 3:**
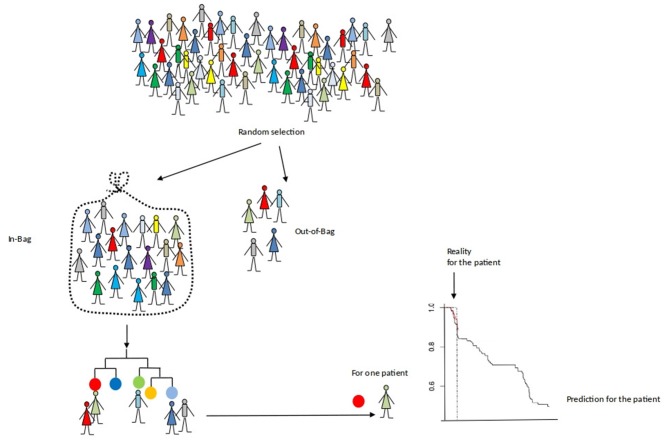
An evaluation of the prediction accuracy of a survival forest.

Figure 3 illustrates this process for the out-of-bag subject (in red).

To estimate the prediction error of our procedure, we use the Harrell's concordance index for survival data or C-index (17). This estimate is widely used in the literature as a way for assessing predictive accuracy in survival analysis. The Harrell's index estimates the probability that, in a randomly selected pair of cases, the case that fails first had a worst predicted cumulative hazard risk estimate. In the following, we reported the final error rate which is equal to one minus the C-index. If this value is 0.5 it corresponds to a procedure doing no better than random guessing, whereas a null value indicates perfect prediction.

### Simulation Study

In order to evaluate the predictive performance of the proposed custom-built RSF procedure as compared to the classical RSF procedure, we conducted the simulation study presented just below. We used the classical Logrank statistic as the splitting criterion for both procedures.

In practice, we used the well-known *Primary Biliary Cirrhosis (PBC)* of the liver data set of the Mayo Clinic, which is publicly available in R through the package “randomForestSRC” (9). This data set is widely used in evaluating survival models and contains well-known explanatory variables. This dataset included 17 potential explanatory variables to which we added 50,000 random variables.

More precisely, we retained only the first 312 patients of the PBC data set who participated in the randomized trial and categorized continuous covariates using their quartiles. Then, we added 50,000 non-informative variables to the PBC dataset. The noise variables were 50,000 pseudo three-genotypes variables obtained from bi-allelic markers in Hardy-Weinberg equilibrium where each pseudo-minor allele is drawn from a uniform distribution ranging between 0.25 and 0.4.

The variable of interest was the time from the start of registration to death. Patients alive were censored at the date of their last follow-up visit. In total, we had 312 individuals with 50,017 candidate variables.

We compared the predictive performance of the classical RSF and the proposed custom-built RSF procedures with a random subsampling ranging from 1 to 100% and measured their error rates. We also computed the error rate obtained for a Bagging survival forest (a special case of RSF with all the covariates are considered as candidate) where the 17 variables were candidates.

In this study, we have considered the classical default parameters for generating trees. Thus, we put no constraint on the depth of the trees but there is a minimal number of unique cases in a terminal node which is of 15. For the RSF, the default parameter for the number of variables randomly selected as candidates for splitting a node is $\sqrt{n}$ where *n* equals the number of candidate variables.

In all cases, the size of the ensemble was fixed at 500 trees.

## Results

###  Simulation Results

**Figure 4:**
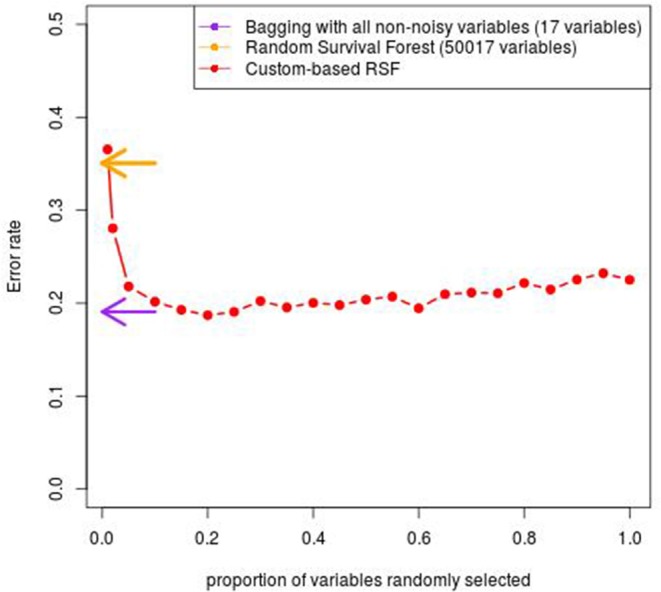
Error rate obtained with the custom-based RSF for various proportion of subsampling.

As seen in Figure 4, We observe that the minimal values of error rate associated with the proposed custom-based RSF procedure is around 19.0%, which is obtained for a proportion of at least 20–25% for the sub-sampling. This value is quite close to the value obtained with the Bagging procedure applied to the small data set containing only the 17 covariates potentially associated with the outcome (18.9%). In comparison, the classical RSF procedure leads to an error rate of 35.1%.

###  ABIRISK Cohort

The cohort analyzed in this work consists in 457 individuals with genotyping information that successfully passed the quality-control procedures and who are suffering from auto-immune diseases. In this multi-cohort, 132 patients (29%) suffered from inflammatory bowel diseases (Crohn's disease or ulcerative colitis), 141 (31%) from multiple sclerosis, and 184 (40%) from rheumatoid arthritis.

There were 310 women (68%) and 147 men (32%). Patients were aged from 18 to 87 years old and the mean age was 43.5 years old. Among the 450 patients for whom the measure was provided, the body mass index (BMI) ranged from 15 to 49 with a mean BMI of 25. Nineteenth patients (4%) were underweighted (BMI <18.5), 252 (56%) had a normal BMI (between 18.5 and 25), 102 (23%) were overweighted (BMI between 25 and 30) and 77 (17%) were obese (BMI > 30). Among the 455 patients with a provided tobacco smoking status, 257 (56%) were currently smoking or had quit smoking and 198 (44%) had never smoked. Among the 445 patients with the provided information, 208 (47%) were taking immunosuppressants during the study. Ninety-four (21%) of the 442 patients whose information was available were taking antibiotics during the study.

Eight biotherapies were used in the study, forming four classes of drugs : TNF-inhibitors (Adalimumab, Etanercept, Infliximab), IFNβ (IFNβ-1a subcutaneous, IFNβ-1a intra-muscular and IFNβ-1b subcutaneous), anti-IL6R (Tocilizumab), and anti-CD20 (Rituximab). 254 patients (55%) were taking TNF-inhibitors, 141 (31%) IFNβ, 35 (8%) anti-IL6R, and 27 (6%) anti-CD20.

In the entire cohort, the probability of producing ADA at 1 year was 27.5% [22.9–31.7%].

###  Prediction Results

We applied our proposed modified RSF procedure on the dataset from the ABIRISK cohort and reported the error rate estimate (one minus the C-index). We also reported the results obtained using the classical RSF procedure [implemented in the “randomForestSRC” package (9)] with default parameters. For both RSF procedures, we ran 500 survival trees. For the modified RSF procedure, each tree-based classifier was grown from random subspaces composed of a subsampling of 75% of the candidate variables.

**Figure 5:**
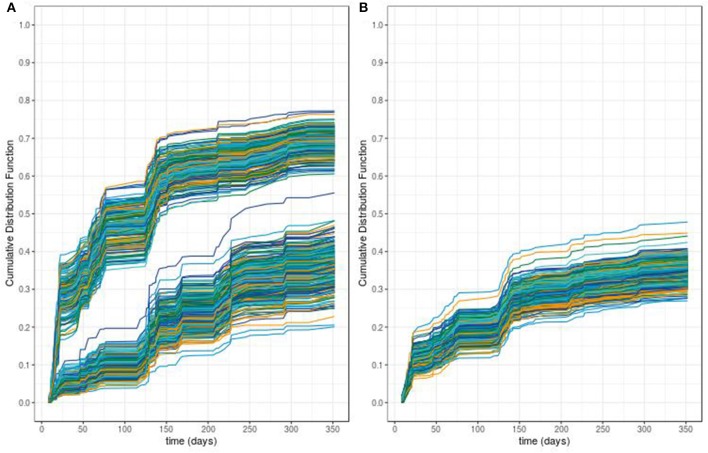
Predicted survival probabilities obtained with our proposed random survival forest method **(A)** vs. those obtained by the classical random survival forest method **(B)**.

The predictive accuracy of our procedure leads to an global error rate of 26.4% whereas the classical RSF leads to a higher global error rate of 51%. From these two procedures, we can compute the out-of-bag individual predicted probabilities of ADA occurrence. Figure 5 displays the predicted cumulative density function for ADA occurrence for the 457 patients obtained using an out-of-bag estimator from both procedures. From this figure, we can see that the modified RSF procedure leads to a clear separation between two groups of individuals regarding the risk of ADA (Figure 5A). Individuals with a predicted probability of occurrence of ADA at 1 year greater than 50% can be considered at high risk whereas those lower than 50% can be considered at low risk. In contrast, the classical RSF procedure was unable to separate individuals regarding the risk of ADA (Figure 5B).

## Discussion

The main contribution of this work is to propose and evaluate the practical use of a custom-built machine learning procedure for time-to-event prediction which accommodates high-dimensional predictors and mixed population of individuals. This was indeed a real issue for evaluating immunogenicity prediction using the bio-clinical data collected from the ABIRISK cohort. The proposed custom-built (or modified) RSF procedure uses a particular splitting criterion and considers random subsamplings of the candidate predictors.

When applying this procedure to the ABIRISK cohort, the modified RSF procedure leads to a much lower error rate than the classical RSF. Moreover, the individual predicted probabilities of ADA occurrence provide a way of discriminating between low and high-risk group of individuals. We can hypothesize that this gain in predictive performance is mainly due to the existence of a tiny proportion of pertinent variables that interact with each other. Indeed, our proposal borrows predictive strength from the tree-based structure while avoiding the restrictive sampling of the classical RSF. Moreover, the counter-performance of the classical RSF shows that there is no one-size-fits-all solution for complex clinical issue, therefore advocating for custom-based solution. In practice, with a random subspace sampling, we ensure that relevant predictors are given to the survival tree growing algorithm as iterations proceed more often than in the classical RSF method, and hence it decreases the chance to include useless survival trees in the forest. From these results, the use of the classical survival random forest should be avoided when few pertinent predictors are expected. Surprisingly, few works have pinpointed this problem that can be however of high concerns for clinical research that copes with so-called fat dataset (i.e., more predictive candidate variables than individuals). It is worth noting that the rationale for considering such multi-diseases/multi-drug predictive analysis was that even though these auto-immune diseases encompass a broad range of phenotypic manifestations, the way the patients respond to various biotherapies suggest that similar clinical and/or biological pathways might be involved and that they also might share some genetic markers. Thus, such cross diseases/drugs strategy should provide gains in predictive power as compared to separate analyses since it borrows information across several therapies and auto-immune diseases. However, if our assumption of common pathways is not correct, we should perform separate analyses (by disease or drugs) that require more individuals to ensure sufficient predictive power.

Despite these promising results, some limitations of the present exploratory predictive study should be considered. First, we should emphasize that our work is only a developmental study (model development and internal validation) which requires further validation studies. Moreover, even if the ABIRISK cohort includes hundreds of participants, it is still a small sample size and future studies with larger cohorts should be done to validate these results and generalize the use of this approach. Moreover, the definition of positivity relies upon the ADA detection methods that were harmonized but however different across the drugs. Second, it is difficult to identify the optimal percentage of subsampling. From our simulation study with fifty thousands of non-relevant variables, we have seen that sampling one-quarter of the feature space seems sufficient for obtaining a good predictive accuracy. For studies with dense genotyping data, we recommend building trees on more than half of the predictors for reaching competitive accuracy. More works should be however performed for providing practical guidance. It is worth noting that including all the predictors such as in the classical bagging strategy can lead to a small increase of the error rate due to the low diversity of the forest. Third, in this work, we use a particular splitting criterion which takes into account the mixture population under study. The choice for this survival model stems from immunological as well as statistical considerations but other modelings could obviously be considered and evaluated. Fourth, this work is only a preliminary step to find new machine learning approaches and further works should be done to derive measures adapted to this approach for identifying the important predictors and decipher their interplay.

In conclusion and to our best knowledge, this is the first study to evaluate the use of machine learning procedures to predict biotherapy immunogenicity based on bioclinical information. We have showed that this custom-based machine learning approach provides a valuable tool for prediction. While the current approach obviously needs further improvement before its clinical practical use, it might have potential to provide useful information for the clinical practice of stratifying patients before giving them a biotherapy.

## Data Availability Statement

The data analyzed in this study were collected in the context of the ABIRISK project by ABIRISK partners. Access to the minimal dataset underlying the findings can be obtained by interested researchers upon request to the ABIRISK Sustainability Scientific Committee by submission of an analysis plan. The analysis plan should explain the purpose of the use of the data and confirm the intention to use the data only for replication studies concerning anti-drug inhibitors, since this is the limitation of the ethical permission on how this data can be used. The contact person of the ABIRISK Sustainability Scientific Committee to whom the requests should be sent is Marc Pallardy (marc.pallardy@inserm.fr).

## Ethics Statement

The studies involving human participants were reviewed and approved by Medical Ethics Committee of the General University Hospital in Prague (reference 125/12, Evropský grant 1.LF UK-CAGEKID) Institutional committee of Heinrich Heine University, Düsseldorf, Germany (protocol reference 4451) Ethikkommission der Fakultät für Medizin der Technischen Universität München, München, Germany (reference 335/13) Ethikkommission Nordwest- und Zentralschweiz, Basel (reference 305/13) Ethikkommission der Medizinischen Universität Inns- bruck, Innsbruck (reference AN2013-0040 331/2.1) Comité Ético de Investigación Clìnica de l'Hospital Universitari Vall d'Hebrón, Barcelona [reference EPA(AG)66/2013(3866)] Stockholm Regional Ethics Committee, Stockholm (reference Dnr. 2013/1034-31/3 and Dnr. 2015/749-32) Comité de Protection des Personnes Ile de France VII (reference 13-048) Medical Ethical Committee of the Academisch Medisch Centrum, Amsterdam (reference 2013-304#B20131074) Local Ethics Committee of AOU Careggi (reference Protocol N o 2012/0035982) NRES Committee London, City & East (reference 14/LO/0506) Comité de Protection des Personnes Ile de France IV (reference 2013/24) Comité d'éthique hospitalo-facultaire universitaire de Liège (reference 2015/55). The patients/participants provided their written informed consent to participate in this study.

## Author Contributions

JD and PB developed the proposed statistical procedure. PB coordinated the project and is JD's Ph.D. thesis advisor. JD and PB participated in writing the original draft. JD, SH, DB, and PB analyzed the data. SH, DB, MA, FD, AF-H, AG, SH-B-A, XM, MP, and PB participated in the collection of the data. MP coordinated the ABIRISK project. All authors read and approved the final manuscript.

### Conflict of Interest

The authors declare that the research was conducted in the absence of any commercial or financial relationships that could be construed as a potential conflict of interest.
